# The steady-state transcriptome of the four major life-cycle stages of *Trypanosoma cruzi*

**DOI:** 10.1186/1471-2164-10-370

**Published:** 2009-08-07

**Authors:** Todd A Minning, D Brent Weatherly, James Atwood, Ron Orlando, Rick L Tarleton

**Affiliations:** 1 Center for Tropical and Emerging Global Diseases, University of Georgia, Athens, Georgia, 30602, USA; 2 Complex Carbohydrate Research Center, University of Georgia, Athens, Georgia 30602, USA

## Abstract

**Background:**

Chronic chagasic cardiomyopathy is a debilitating and frequently fatal outcome of human infection with the protozoan parasite, *Trypanosoma cruzi*. Microarray analysis of gene expression during the *T. cruzi *life-cycle could be a valuable means of identifying drug and vaccine targets based on their appropriate expression patterns, but results from previous microarray studies in *T. cruzi *and related kinetoplastid parasites have suggested that the transcript abundances of most genes in these organisms do not vary significantly between life-cycle stages.

**Results:**

In this study, we used whole genome, oligonucleotide microarrays to globally determine the extent to which *T. cruzi *regulates mRNA relative abundances over the course of its complete life-cycle. In contrast to previous microarray studies in kinetoplastids, we observed that relative transcript abundances for over 50% of the genes detected on the *T. cruzi *microarrays were significantly regulated during the *T. cruzi *life-cycle. The significant regulation of 25 of these genes was confirmed by quantitative reverse-transcriptase PCR (qRT-PCR). The *T. cruzi *transcriptome also mirrored published protein expression data for several functional groups. Among the differentially regulated genes were members of paralog clusters, nearly 10% of which showed divergent expression patterns between cluster members.

**Conclusion:**

Taken together, these data support the conclusion that transcript abundance is an important level of gene expression regulation in *T. cruzi*. Thus, microarray analysis is a valuable screening tool for identifying stage-regulated *T. cruzi *genes and metabolic pathways.

## Background

*Trypanosoma cruzi*, the etiologic agent of Chagas disease in humans, is a protozoan parasite which assumes four morphological stages during its cycle in insect and mammalian hosts. In the reduviid insect vector, *T. cruzi *epimastigotes replicate extracellularly in the lumen of the gut. As the parasite stages reach the posterior end of the gut they attach to the wall of the rectum and convert to non-replicative, infective metacyclic trypomastigotes that are released in the feces when the bug takes a blood meal. The metacyclic trypomastigotes enter the mammalian host when they are rubbed into the bite wound or other open skin or mucosal surfaces and invade host cells. The intracytoplasmic parasites convert to the replicative, amastigote stage and undergo many rounds of division before transforming into elongated, motile trypomastigote stages which are released when the host cell ruptures. The trypomastigotes are disseminated in the blood and lymph where they may infect virtually any nucleated cell or be taken up by the insect vector to complete the life cycle. A detailed description of the *T. cruzi *life-cycle with accompanying diagram can be accessed at the web site for the Centers for Disease Control and Prevention http://www.dpd.cdc.gov/dpdx/html/trypanosomiasisamerican.htm.

There are no vaccines for Chagas disease, and specific chemotherapy for *T. cruzi *infection is hindered by adverse side-effects and questionable efficacy. In order to broadly screen for genes with relevant expression patterns, and to learn more about the biology of *T. cruzi *in general, we and others have conducted transcriptomic and proteomic analyses of *T. cruzi *life-cycle stages [1-11]. Although these analyses have highlighted potential drug and vaccine targets, they have been somewhat limited in scope, largely non-quantitative, and rarely correlated transcript and protein abundances. The latter issue is of particular importance for *T. cruzi *and other kinetoplastids, due to the generally accepted view that regulation of gene expression in the kinetoplastids is almost entirely post-transcriptional (reviewed in [12,13]). In fact, only one kinetoplastid RNA pol II promoter has been described despite repeated attempts in many laboratories (reviewed i [14]). Other studies have implicated mRNA processing [15], translational repression [16-18], polysome recruitment [19], and codon adaptation [20] in the regulation of gene expression in the kinetoplastids, all processes that would be predicted to mitigate the role of mRNA abundance regulation in determining protein expression levels. Indeed, previous microarray studies in the kinetoplastids have revealed relatively modest numbers of genes whose transcript abundances are significantly regulated (reviewed in [21]). There is, however, plentiful evidence that transcript levels in these parasites are controlled by mRNA decay, involving 3'-UTRs and RNA-binding proteins [19,22-28]. To determine the extent of mRNA abundance regulation in *T. cruzi *globally, we performed whole-genome, DNA microarray analysis of the four life-cycle stages of *T. cruzi*. The results of these analyses were compared with existing protein expression data for *T. cruzi *to determine the correlation, if any, between transcript and protein relative abundances in this human pathogen.

## Results

### *T. cruzi *displays significant stage-regulation of relative transcript abundances for thousands of its genes

**Figure 1:**
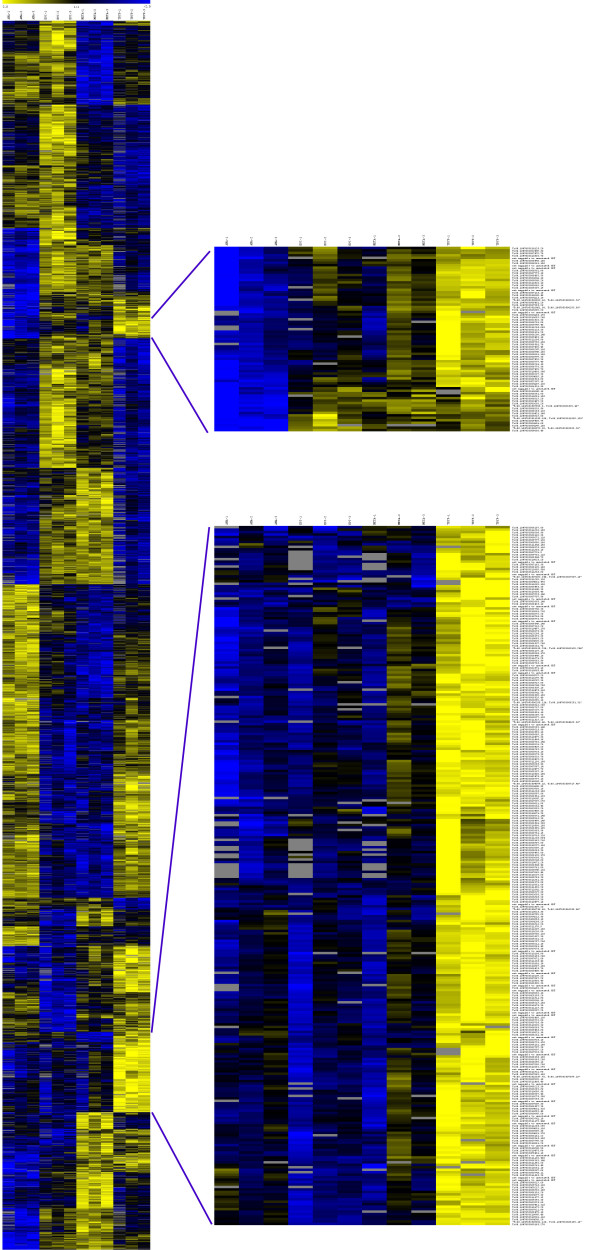
**Heat map of genes significantly regulated during the life-cycle of *Trypanosoma cruzi***. Ratios are log2 (stage/reference), thus yellow bars represent upregulation and blue bars represent downregulation. Two trypomastigote upregulated clusters are expanded. The spots annotated as 'not mappable to annotated ORF' were designed from earlier versions of the *T. cruzi *genome sequence and subsequently designated as 'obsolete' by TIGR. These spots no longer mapped within an annotated ORF in the final version of the genome sequence, e.g. they were in intergenic regions (such as untranslated regions) and/or were antisense (would hybridize with first strand cDNA from transcripts from the non-coding strand).

Co-hybridization of cDNAs from each *T. cruzi *life-cycle stage with a reference cDNA sample comprised of all four life-cycle stages on oligonucleotide, whole genome microarrays, revealed that over 83% of the oligonucleotides detected transcript above background levels and were consistent between dye-swap replicates in at least three life-cycle stages (10,256/12,288). Significance Analysis of Microarrays (SAM) [29] determined that a total of 4,992 of these transcripts exhibited statistically significant up or down regulation in at least one of the four life-cycle stages with a median false discovery rate (FDR) of 0.06071% (3.03 spots) and a 90th percentile FDR of 0.40582% (20.26 spots). Whether one includes or excludes members of large gene families from the analysis, >50% of the genes with detectable signals were significantly regulated during the course of the *T. cruzi *life-cycle (Table 1). The significant regulation of thousands of *T. cruzi *genes is visually apparent in the heat map of significantly regulated genes (Figure 1). Note that the significantly regulated genes show consistency between biological replicates, particularly in stages where the extent of up or down regulation is the greatest. The expanded clusters in Figure 1 are highly enriched in trypomastigote-upregulated trans-sialidases (TS) and mucins.

**Table 1:** With or without the contribution of large gene family members, greater than 50% of the *Trypanosoma cruzi *genes detected on whole-genome, oligonucleotide microarrays were significantly stage-regulated at the RNA level.

		# spots (percent of total)	# genes (percent of total)
all	SIG^1^	4273 (51.6)	6708 (52.2)
	NON-SIG^2^	4000 (48.4)	6141 (47.8)
	**Total**	**8273 (100)**	**12849 (100)**

large gene families^3^	SIG	739 (50.1)	1010 (51.5)
	NON-SIG	735 (49.9)	950 (48.5)
	**Total**	**1474 (100)**	**1960 (100)**

minus large gene families	SIG	3534 (52.0)	5698 (52.3)
	NON-SIG	3265 (48.0)	5191 (47.7)
	**Total**	**6799 (100)**	**10889 (100)**

The oligonucleotide sequences were aligned against the *T. cruzi *genome by BLAST analysis [58]. Oligonucleotides showing 80% or greater identity with more than three *T. cruzi *gene ID's were not counted.

^1^Significantly stage-regulated as determined by SAM analysis (FDR = 0.4%).

^2^Non-significantly stage-regulated as determined by SAM analysis (FDR = 0.4%).

^3^Oligonucleotides mapping to members of large gene families (number of members > 30).

Of the genes upregulated in two stages, 76% (1083/1423), were co-upregulated in stages occurring in the same host (mammalian hosts – amastigotes and trypomastigotes; insect hosts – epimastigotes and metacyclic trypomastigotes), whereas approximately 20% (282/1423) were co-upregulated in stages with similar biological functions. For example, genes co-upregulated in amastigotes and epimastigotes (dividing stages) were enriched in those involved in DNA repair, including proliferative cell nuclear antigen (PCNA). Likewise, genes co-upregulated in trypomastigotes and metacyclic trypomastigotes (non-dividing, infective stages) included never in mitosis (NIMA) related kinase and were enriched for trans-sialidases (TS), which are involved in invasion (reviewed in [30]).

### The validity of *T. cruzi *microarray data is supported by qRT-PCR and by comparison to existing proteomic data

The accuracy of the microarray results was confirmed by quantitative reverse-transcriptase PCR (qRT-PCR) for a subset of the significantly regulated genes.

**Figure 2:**
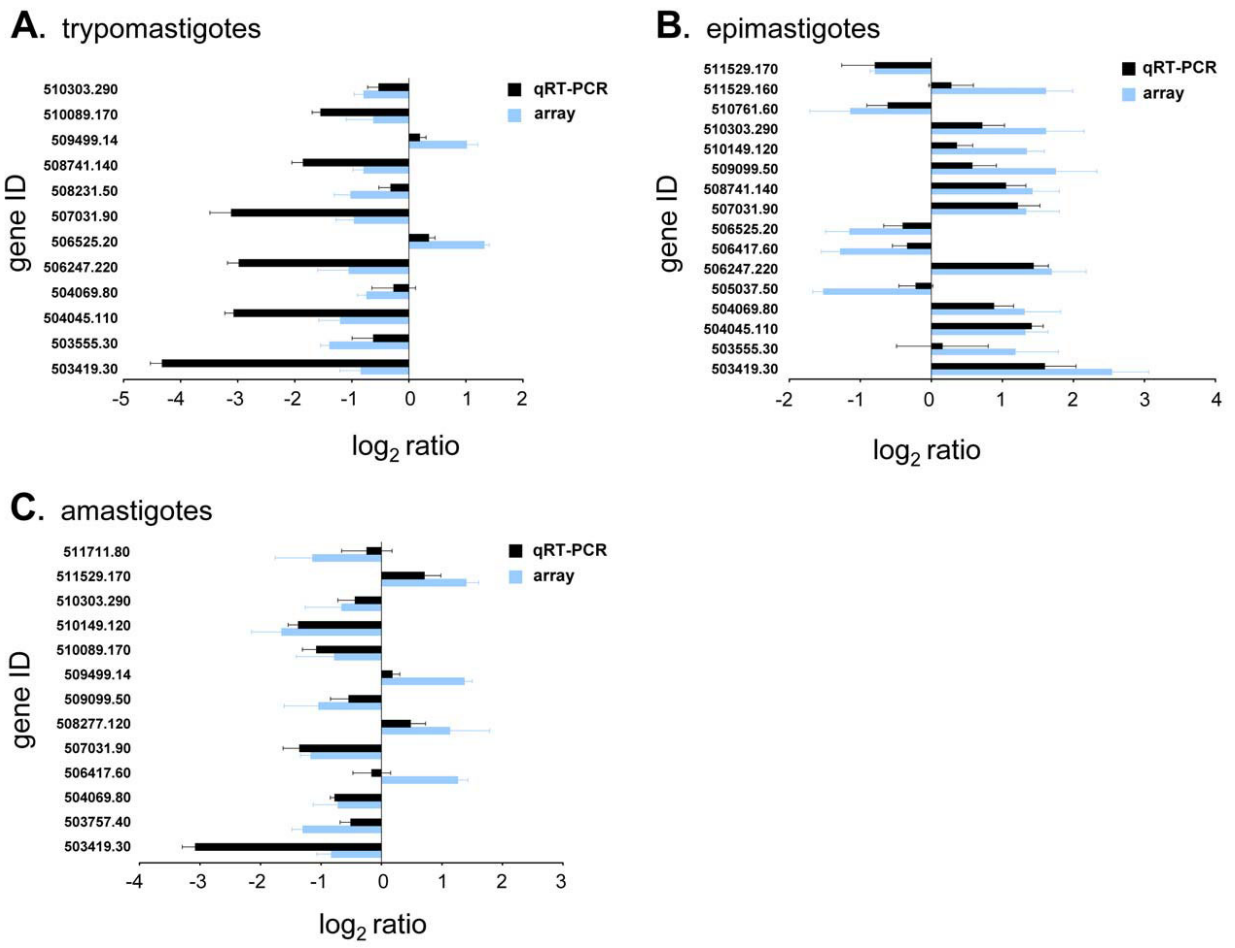
**qRT-PCR confirmation of relative transcript abundances for microarray-identified, significantly regulated genes**. Mean log_2 _ratios (stage/reference) for selected genes in selected stages determined by qRT-PCR and microarray analyses. Gene ID's are official gene symbols (prefix Tc00.1047053 is truncated) assigned to annotated genes in the reference CL Brener *T. cruzi *genome. Error bars are standard deviations for three biological replicates for both the microarray and qRT-PCR data. The qRT-PCR and microarray standard deviations reflect biological, not technical variation. A) trypomastigotes B) epimastigotes C) amastigotes.

For 12 out of 12 genes showing significant up or downregulation by microarray analysis in trypomastigotes, qRT-PCR data agreed in direction of regulation (Figure 2A). Similar agreement was seen in the case of epimastigotes (16 out of 16) and amastigotes (13 out of 14) genes (Figure 2B and 2C).

**Figure 3:**
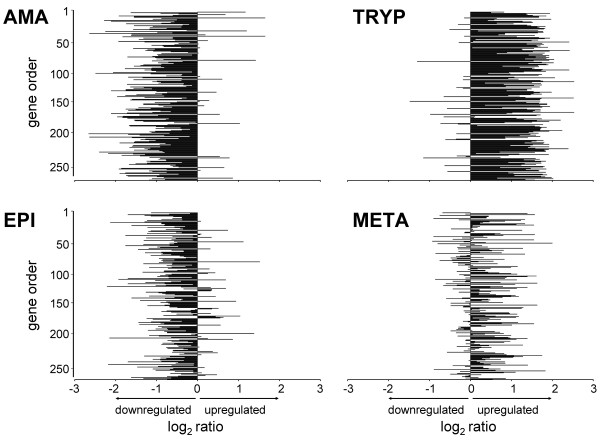
**Microarray-identified, significantly regulated *Trypanosoma cruzi *trans-sialidase (TS) genes**. Shown are mean microarray log_2 _ratios (stage/reference) for TS significantly regulated in *Trypanosoma cruzi *amastigotes (AMA), trypomastigotes (TRYP), epimastigotes (EPI), and metacyclic trypomastigotes (META). The y-axis scale is provided as a reference for gene order for the genes in Additional file 8, which shows the mean log2 ratios for three biological replicates for each life cycle stage and their standard deviations. The genes are in the same order in each panel.

**Figure 4:**
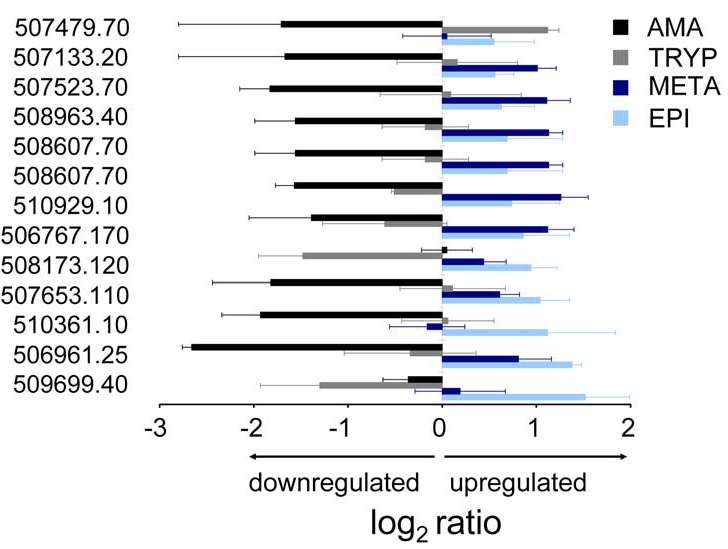
**Microarray-identified, epimastigote upregulated *Trypanosoma cruzi *trans-sialidase (TS) genes**. Mean microarray log_2 _ratios (stage/reference) in amastigotes (AMA), trypomastigotes (TRYP), epimastigotes (EPI), and metacyclic trypomastigotes (META) for significantly regulated TS that were at least two-fold upregulated in epimastigotes. Gene ID's are official gene symbols (prefix Tc00.1047053 is truncated) assigned to annotated genes in the reference CL Brener *T. cruzi *genome. Error bars are standard deviations for three biological replicates and reflect biological, not technical variation.

Further validation of the microarray results came from comparison to known protein expression profiles for selected genes. For example, proteomic analyses indicated most TS gene expression occurs in trypomastigotes and significantly fewer TS proteins are detected in amastigotes and metacyclic trypomastigotes (and none detected in epimastigotes) [8]. A similar pattern of expression was evident in the transcriptome data (Figure 3 and Additional file 8). Gene set enrichment (GSE) analysis [31,32] of genes >2-fold upregulated in trypomastigotes and significantly regulated (by SAM analysis) also revealed enrichment of trans-sialidases (using the gene ontology (GO) molecular function term "exo-alpha-sialidase activity"; hypergeometric P-value of 4.1E-25; 1.8E-22 with Benjamini correction for FDR). Several TS mRNAs were detected in epimastigotes, in agreement with previous EST analyses [33]. Interestingly, of the 12 TS genes showing 2-fold or greater transcript upregulation in epimastigotes, 11 were also upregulated in metacyclic trypomastigote stages (Figure 4), consistent with the possibility they are translationally repressed in epimastigotes and subsequently translated in metacyclic trypomastigotes.

**Figure 5:**
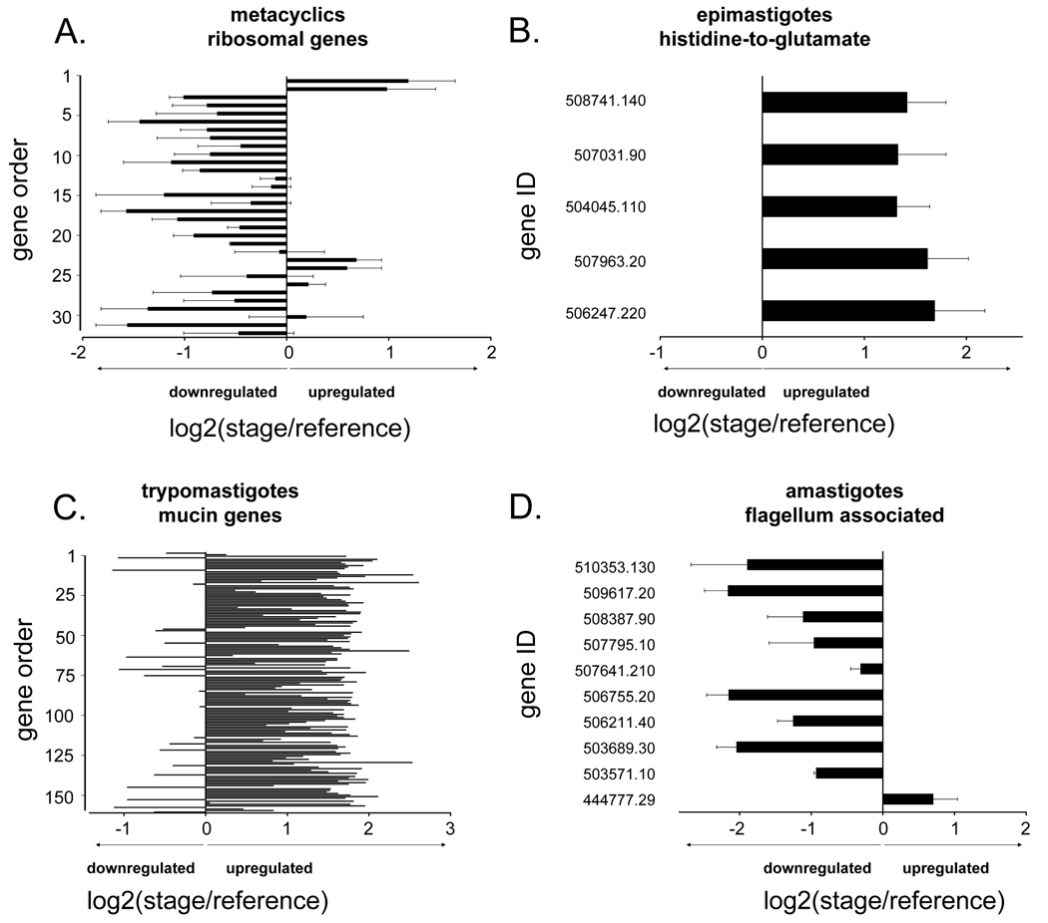
**Transcript relative abundances for selected functional groups agree with published protein expression data**. Shown are mean transcript log_2 _ratios (stage/reference) from *T. cruzi *oligonucleotide microarray analyses for A) ribosomal protein genes in metacyclic trypomastigotes, B) genes in the histidine-to-glutamate pathway in epimastigotes, C) mucin genes in trypomastigotes, and D) flagellum-associated genes in amastigotes. The ribosomal genes were selected based on their having the term 'ribosomal' in their annotation and their having either proteome or transcriptome data associated with them. Gene ID's in panels B and D are official gene symbols (prefix Tc00.1047053 is truncated) assigned to annotated genes in the reference CL Brener *T. cruzi *genome. In panels A and C the y-axis scale is provided as a reference for gene order for the genes in Additional files 9 and 10, respectively, which show the mean microarray ratios for three biological replicates for each life cycle stage and their standard deviations. Error bars in panels A, B, and D are standard deviations for three biological replicates and reflect biological, not technical variation.

Other gene groups showing concordance between the transcriptome and whole proteome analyses included genes for ribosomal proteins (downregulated in metacyclics) and genes in the histidine-to-glutamate pathway (upregulated in epimastigotes) [8] (Figure 5A and 5B and Additional file 9). We also observed the expected upregulation of mucin genes in trypomastigotes (reviewed in [34]; confirmed by hypergeometric P-value of 1.5E-37 for GSE) and the downregulation of flagellum-associated genes in amastigotes (which lack flagella; hypergeometric P-value of .00153 for GSE) (Figure 5C and 5D and Additional file 10).

### The *T. cruzi *microarray data provide expanded coverage of important functional groups

The concordance between the presently reported transcriptomic data and published proteomic data suggests that the mRNA relative abundances observed here will be useful indicators of the protein expression levels of genes for which protein expression data has not been reported, including many genes annotated as 'hypothetical.' Current proteomic methods often fail to detect or quantify proteins that are expressed at low levels, (e.g. protein kinases and phosphatases; reviewed in [35]) or proteins that have physical properties making them less amenable to proteomic analyses, (e.g. membrane proteins; reviewed in [36]). For example, this transcriptomic analysis detected 30 protein phosphatases and 128 protein kinases, which were significantly regulated at the transcript level (Additional files 1 and 2), and few of which have published protein expression data. Likewise, our transcriptome analysis detected 166 significantly regulated genes annotated as 'integral-to-membrane' (Additional file 3).

### Members of many *T. cruzi *paralog clusters exhibit significant stage-regulated divergence in mRNA relative abundance patterns

**Figure 6:**
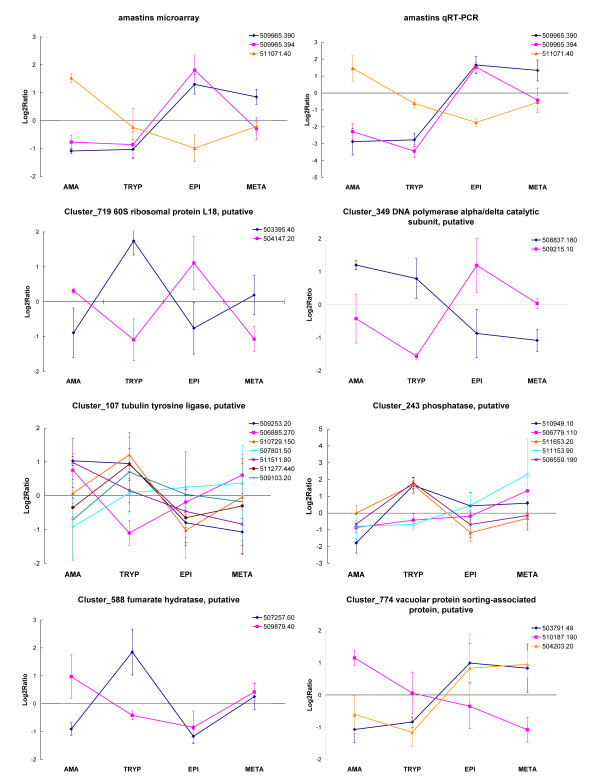
**Duplicate genes in *Trypanosoma cruzi *show divergent expression patterns**. Microarray expression data for paralog groups containing 3–15 members were compared to identify groups showing divergent expression between duplicate genes. Shown are selected groups with representative divergent expression patterns. Gene ID's are official gene symbols (prefix Tc00.1047053 is truncated) assigned to annotated genes in the reference CL Brener *T. cruzi *genome. Expression data for the amastins were confirmed by qRT-PCR (top right panel). Error bars are standard deviations for three biological replicates for both the microarray and qRT-PCR data. The qRT-PCR and microarray standard deviations reflect biological, not technical variation.

Kinetoplastids appear to maintain multiple copies of genes for which high level expression is required [37,38]. Hence we expected that genes in paralog clusters would have similar expression patterns and were surprised to find that over 10% (105/1023) of the *T. cruzi *paralog groups containing 3–15 members had divergent mRNA relative abundance patterns (Figure 6). One of these groups was the amastins, a paralog group previously reported to be largely amastigote-specific [37] but herein shown to include members with significant stage-specific upregulation in insect stages. The divergent expression patterns of the *T. cruzi *amastins were confirmed by qRT-PCR (Figure 6) and are also supported by the detection of amastin in the metacyclic trypomastigote proteome [8].

In addition to increasing transcript abundance, another presumed function of gene duplication is to allow for the development of new protein functions [38]. Such neofunctionalization may have occurred in the *T. cruzi *60S ribosomal L18 protein paralog group (Figure 6). Tc00.1047053503395.40, which was upregulated in trypomastigotes, has a 130 amino acid N-terminal extension not found in any other publicly-available 60S ribosomal L18 sequence, suggesting the possibility that this paralog has a novel function in *T. cruzi *trypomastigotes.

## Discussion

Transcriptomic analyses have not been as aggressively pursued in kinetoplastid organisms as they have been in other protozoan parasites, such as *Plasmodium*. This is likely due to the view that the predominantly post-transcriptional nature of gene expression regulation in kinetoplastids makes microarray studies in these organisms generally less informative than microarray studies in organisms for which transcription initiation plays a larger role in gene expression regulation. In support of this, the ranges of observed mRNA ratios between life-cycle stages in kinetoplastid organisms have been relatively narrow compared to the ranges observed in organisms with canonical RNA pol II promoters. For example, 1,000-fold induction in relative transcript abundance for CD70 was observed in a microarray study comparing Human T-lymphotropic virus type 1 (HTLV-1) carrying T-cell lines versus HTLV-1-negative T-cell lines [41], whereas in kinetoplastid microarray studies, fold inductions above ~8-fold are rarely observed [3,42-46].

Further contributing to the debate over the utility of microarray studies in kinetoplastids has been the general observation that relatively few genes have exhibited significant stage regulation of mRNA relative abundances in previous microarray studies. For instance, Diehl et al., using arrays of 21,024 PCR-amplified genome shot-gun library inserts from *T. brucei *to compare relative transcript abundances between *in vitro *cultured human, long slender forms and procyclic parasites, observed that, although 75% of the array elements detected transcripts, only 2% displayed significant differences between the two life-cycle stages [39]. The observation that only 2% of the detected transcripts differed significantly in relative abundance between two very different life-cycle stages supported the concept that transcript abundances in *T. brucei *are largely constitutive, especially considering the two life-cycle stages were compared directly on the microarrays. This picture of predominantly constitutive genome expression at the level of transcript abundance in kinetoplastids was reinforced manifold by subsequent studies in *T. brucei *[40] and *Leishmania spp*. (reviewed in [41]).

In light of the findings from *T. brucei *and *Leishmania spp*. microarray studies as well as findings from our previous, limited microarray study in *T. cruzi *[3], we sought to determine if transcript levels are globally variable between life-cycle stages of *T. cruzi *using whole-genome oligonucleotide microarrays. When cDNAs from each life-cycle stage were co-hybridized with a reference cDNA sample comprised of all four life-cycle stages on oligonucleotide, whole genome microarrays, we observed that the relative transcript abundances for over 50% of the genes detected on the arrays were significantly regulated between the life-cycle stages.

The microarray data for selected genes were validated by qRT-PCR. There was generally good agreement between the two platforms in terms of direction of regulation, however there were some quantitative differences, especially in terms of the extent of up or downregulation estimated by these two techniques. These quantitative differences likely were the result of sequence-specific effects and differences in dynamic range for the two platforms [49,50]. In addition, the RNA samples used for qRT-PCR were not the identical samples used for the microarrays.

Recent improvements in microarray design, such as the use of oligonucleotide probes designed for more uniform hybridization kinetics and with lower likelihood of cross-hybridization than amplicon-derived probes and the use of microarrays with greater genome coverage could in part account for why our study identified a higher percentage of significantly stage-regulated genes than previous microarray studies in other kinetoplastids [1-4,39,42]. However, even a recent study by Rochette et al. that used *Leishmania *whole genome oligonucleotide microarrays to directly compare amastigotes and procyclics reported that only 7% of the genes in *Leishmania infantum *and 9.3% of the genes in *L. major *were developmentally regulated [43]. It is also possible that the use of different statistical methods for analysis of microarray data my yield widely different estimates of the frequency of regulated genes. However, using less rigorous statistical criteria for determining significance, such as fold-change, results in higher false discovery rates [29] and thus would be predicted to overestimate the number of significantly regulated genes. Our reanalysis of the Papadopoulou data from *L. infantum *using our statistical methods found 715 genes (7% of the genes fulfilling our criteria for detection) to be significantly regulated between amastigotes and promastigotes with an FDR of 1.55% (an FDR nearly 4 times greater than what we report for *T. cruzi*). Thus, different statistical analysis methods cannot account for the differential estimates of the frequency of regulated genes in *Leishmania *and *T. cruzi*.

By incorporating all four life cycle stages of *T. cruzi *in our analysis, in contrast to the 2 stage comparisons done with other kinetoplastids (e.g. procyclic to amastigote [43] or procyclic to bloodstream forms [39]) we have increased the possibility of identifying a greater number of genes regulated in expression in at least one of the 4 life cycle stages. However, the contribution of increased number of stages studied to the overall number of significantly regulated genes was greatly mitigated by the fact that we used a reference design in which each life-cycle stage was compared to a mixture of all four life-cycle stages. This design minimized the number of microarrays required to compare the four life-cycle stages (each stage vs. reference = 24 hybridizations in the present study, each stage vs. each other stage = 36 hybridizations for equivalent replication), and also simplified the analysis, but had the disadvantage of buffering the ratios observed. By contrast, previous microarray studies in the kinetoplastids involved directly comparing one life-cycle stage with another on the microarrays, an experimental design which should maximize the number of significant ratios. Thus our estimate that >50% of genes in *T. cruzi *have significantly stage-regulated transcript levels is much more likely to be an underestimate than an overestimate.

The number of significantly stage-regulated transcripts reported here for *T. cruzi *is also likely to be underestimated because >2,000 of the oligonucleotide probes detected transcripts in two or fewer stages and were removed from further analysis for statistical reasons (significance determination for such genes would have been dubious, because SAM analysis of four groups (life-cycle stages) was unreliable for genes present in less than three groups). Also, almost certainly some of the genes that we reported as non-significantly regulated are significantly regulated in transitional stages of the life-cycle. For instance, Saxena et al., using microarrays of ~8300 PCR-amplified genome survey sequencing (GSS) clone inserts, identified 344 protein coding genes significantly regulated during axenic promastigote-to-amastigote differentiation in *Leishmania donovani *[52]. Of these, 136 genes, nearly 40% of the total number of differentially regulated genes identified in the study, displayed transient up or downregulation that would have been missed by only comparing fully differentiated parasites. Thus our analysis of only fully differentiated stages likely underestimated the percentage of genes we found significantly regulated at the transcript level during the *T. cruzi *life cycle, suggesting that time course studies looking at transitional stages in *T. cruzi *would be fruitful.

The disparity in the degree of transcript abundance regulation between *T. cruzi *and the other sequenced kinetoplastids suggests there may be differences in the number and kind of RNA binding proteins in their genomes, because almost all regulation of mRNA abundance in these organisms is at the level of mRNA stability. However, the genomes of *T. cruzi*, *T. brucei*, and *L. major *all have remarkably similar complements of RNA binding proteins (reviewed in [44]). Of the 77 proteins with RNA Recognition Motifs (RRMs) in *T. cruzi*, *T. brucei*, and *L. major*, only two were unique to *T. cruzi*, RBP4 (Tc00.1047053508901.20) and DRBD8 (Tc00.1047053503709.10, Tc00.1047053509581.50), both of which were upregulated in metacyclic trypomastigotes.

Present knowledge of the kinetoplastids does not elicit an obvious biological explanation for why gene expression regulation in *T. cruz*i might be so different from its closest relatives. Perhaps the most striking difference between *T. cruzi *and *T. brucei *and *L. major *is that only *T. cruzi *has both intracellular and extracellular life-cycle stages in the mammalian host. Amastigotes must replicate intracellularly and, although sequestered from the actions of antibodies and complement, are subjected to attack by CD8+ cytotoxic T lymphocytes (CTL) [45] and to the effects of host cytokines [46] and antimicrobials, including reactive oxygen species and nitric oxide [47]. Trypomastigotes are extracellular and thus must evade host defenses including complement, antibodies, and phagocytes. An enhanced capacity for regulating transcript abundances may have provided *T. cruzi *with the agility necessary to rapidly shift between such different environments.

The finding that *T. cruzi *divergently regulates mRNA relative abundances for members of paralog clusters was unexpected, given the post-transcriptional mode of gene expression regulation in this parasite. Divergent expression of duplicate genes is believed to occur by the accumulation of mutations in non-coding *cis *regulatory sequences, such as promoters [48]. Since kinetoplastids appear to be largely devoid of canonical promoters, it will be interesting to study the 3' untranslated regions of paralogs with divergent expression patterns to identify the sequences responsible for the differential mRNA abundances.

The differentially regulated *T. cruzi *duplicate genes identified here will be useful to study the retention and neofunctionalization of duplicate genes in an ancient eukaryote lacking transcriptional control of gene expression. In such an environment, the contributions of mRNA stability control and sub-cellular localization to the evolution of new genes can be studied in isolation from the effects of transcriptional control. Gene knockout studies are currently underway to determine if the array-identified, divergently-expressed paralogs, such as the trypomastigote upregulated 60S ribosomal L18 protein, have novel functions.

## Conclusion

*T. cruzi *displays stage-regulated control of mRNA abundances for a significant proportion of its genes despite the fact that regulation of gene expression in the kinetoplastids is primarily post-transcriptional and despite the fact that previous genomic studies of the kinetoplastids have found that only a low proportion of their genomes are stage-regulated. Moreover, *T. cruzi *oligonucleotide microarray measurements largely agree with known protein expression data for key functional groups. Taken together, *T. cruzi *oligonucleotide microarray analysis is a useful screening tool to determine stage-regulated gene expression in this human pathogen.

## Methods

### Parasites

Brazil strain *T. cruzi *trypomastigotes were grown in monolayers of Vero cells (ATCC no. CCL-81) in RPMI supplemented with 5% horse serum as previously described [49]. Emergent trypomastigotes were harvested daily and examined by light microscopy to determine the percentages of amastigotes and trypomastigotes. Only preparations containing > 95% trypomastigotes were used in the subsequent studies. Amastigotes were prepared from axenically-induced trypomastigotes as described previously [50]. Briefly, emergent trypomastigote samples were centrifuged at 3,000 × g for 15 m at room temperature, brought to density of 5 × 10^6^/ml in Protein-Free Hybridoma Medium (PFHM-II, Gibco, Bethesda, MD) at pH 5.0, and incubated at 37°C until greater than 95% of the parasites were fully converted to the amastigote stage as determined by microscopic examination. *T. cruzi *epimastigotes were grown in Liver Infusion Tryptose media (LIT) as previously described [51]. Cultures were harvested during mid-log phase by centrifugation at 3,000 × g for 10 m at room temperature. Metacyclic trypomastigotes were obtained from epimastigotes by axenic induction as previously described [52]. Briefly, log phase epimastigote cultures were centrifuged at 3,000 × g for 15 m at room temperature, brought to a density of 5 × 10^6^/ml in Complete Grace's Insect Medium (Sigma no. G8142, Sigma, St. Louis, MO) supplemented with 10% fetal bovine serum, pH 6.6, and incubated at 29°C for 10–14 d. The percentages of metacyclics were determined by microscopic examination of parasites stained with Dif-Quick (Baxter Diagnostics, McGaw Park, IL).

### Preparation of labeled cDNAs

Total RNA was isolated from parasites with the High Pure Total RNA Isolation Kit (Roche) per the manufacturer's instructions, including on-column DNase I digestion. Labeled first strand cDNA was synthesized from the total RNA samples using Superscript™ II reverse transcriptase (Life Technologies, Grand Island, NY) as previously described [3]. Briefly, 10 μg RNA were combined with 2 μg oligo-d(T) (Life Technologies), brought to 10 μl with water, denatured at 70°C for 10 m, and cooled on ice. Final 30 ul reactions were assembled with final concentrations of 1× First Strand Buffer (Life Technologies), 10 mM DTT (Life Technologies), 0.5 mM dATP, dCTP, and dGTP (Amersham Pharmacia Biotech, Piscataway, NJ), 0.1 mM dTTP (Amersham Pharmacia Biotech), 0.1 mM Cy3- or Cy5-dUTP (Amersham Pharmacia Biotech), and 13.3 U/μl Superscript II reverse transcriptase (Life Technologies). The reaction mixtures were incubated at 42°C for 2 h and stopped with the addition of 1.5 μL 20 mM EDTA. To degrade the RNA template, 1.5 μL 500 mM NaOH were added and the reactions were heated at 70°C for 10 m. The reactions were neutralized by adding 1.5 μL 500 mM HCl, and unincorporated fluorescent nucleotides were removed using GFX columns per the manufacturer's instructions (Amersham Pharmacia Biotech). The purified products were eluted using 50 μL TE, pH 8.0, and the labeled cDNA was completely dried in a SpeedVac (Savant Instruments, Holbrook, NY, USA) and resuspended in 10 μL water.

### Microarrays

The *T. cruzi *microarrays were obtained from the Pathogen Functional Genomics Resource Center (PFGRC). The microarray description is available at http://pfgrc.tigr.org. The 12,288 unique array oligonucleotides were sense-strand 70-mers designed against open reading frames in the annotated CL Brener reference genome sequence and were printed in duplicate. The microarrays contained an additional 500 control oligonucleotides designed from *Arabidopsis *sequences, also printed in duplicate.

To assure accuracy in the spot-to-gene annotation, the sequences of the oligonucleotides used on the TIGR *T. cruzi *microarrays were remapped onto the *T. cruzi *genome and assigned to genes. The mapping was complicated by the fact that *T. cruzi *is diploid and the CL-Brener strain used for genome sequencing is a hybrid strain [53]. Thus, the two alleles for genes in the *T. cruz*i genome frequently differ by 1–2% in their coding sequences [54], and the terms 'allele' and 'gene' are conflated when describing the *T. cruzi *genome. Moreover, complete chromosomes were never assembled for the *T. cruzi *genome [54], and triploidy for some loci has been confirmed [55]. As a result, the total number of 'genes' identified by the oligonucleotides on the microarrays was greater than the number of unique oligonucleotides. Thus, oligonucleotides mapping with greater than 80% homology to three or fewer 'genes' were used in the subsequent analyses, and oligonucleotides that mapped to more than three genes were not included in the enumeration of significantly regulated genes. Additional file 4 shows the results of our remapping of the oligonucleotides to the *T. cruzi *annotated genome, the original TIGR mapping, and the results of the cross-hybridization prediction. Additional file 5 shows the representation of large gene families (cluster designations per [54]) on the microarrays. The mucin associated surface protein (MASP) family in particular was poorly represented on the microarrays.

### Microarray prehybridization

The microarrays were prehybridized immediately preceding hybridization as described on the PFGRC website. Briefly, arrays are incubated in prehybridization solution (4× SSC, 40% formamide, 0.1% SDS, 0.5% BSA, and 25 mM Tris-HCl, pH 8.0) at 42°C for 1 h. Slides are then washed at room temperature in 0.2% SDS, followed by 3 washes with water, and dried by centrifugation.

### Microarray hybridization

Six hybridizations were performed for each life-cycle stage. The hybridizations consisted of three dye-swap experiments from three independent samples (biological replicates). In each case, the experimental sample was from a single life-cycle stage and the control sample was an equal mixture of all four life-cycle stages. Hybridizations were performed as previously described [3]. Briefly, equal volumes of control and experimental labeled cDNAs were combined with mouse CoT1-DNA (Life Technologies), poly(A)-DNA (Amersham Pharmacia Biotech), and hybridization buffer to yield final concentrations of 4× SSC, 40% formamide, 0.1% SDS, 0.5 μg/μl CoT1-DNA, 0.5 μg/μl poly(A)-DNA, and 25 mM Tris-Cl, pH 8.0). Samples were heat denatured at 95°C for 3 m, centrifuged at 12,000 × g at room temperature for 1 m, applied to the prehybridized array in a MAUI mixer chamber (BioMicro Systems, Inc., Salt Lake City, Utah), placed in a MAUI hybridization apparatus and hybridized at 42°C for 16–20 h with constant mixing. Following hybridization, the array, with attached mixer, was removed from the MAUI apparatus, the mixer was removed from the array, and the array was washed once for 2 m at room temperature in 300 ml PBS with 0.05% SDS, followed by 3 washes for 2 m each at room temperature in 300 ml PBS, and one wash for 1 m at room temperature in 300 ml 0.2× PBS. The slide was then dried by centrifugation.

### Scanning and data analysis

Immediately following hybridization and washing, arrays were scanned on a ScanArray 4000 (GSI Lumonics, Wilmington, MA) at the Integrated Biotech Laboratories at the University of Georgia. Microarrays were scanned at multiple laser and photomultiplier tube (PMT) settings in each channel to obtain Tagged Image File Format (TIFF) images of matching sensitivity in the two channels. Microarray scans were quantified using The Institute for Genomic Research (TIGR) SpotFinder module of TM4 http://www.tm4.org. Individual sub-grids within each scan-specific grid were aligned manually. Background correction was used to flag spots with signal intensities less than local background intensity plus one standard deviation. The Otsu algorithm [56] was used to identify spots. The signal intensity files generated in SpotFinder were imported into the TIGR Microarray Data Analysis System (MIDAS) module of TM4 http://www.tm4.org using default parameters: e.g. MIDAS filtered spots with signal intensity in either channel less than 1, spots with signal intensities less than 2 × local background in either channel, and spots with integrated signal intensities less than 10,000. Since spots typically contained approximately 100 pixels, integrated intensities of 10,000 corresponded to mean signal intensities of 100. Normalization was performed by locally weighted least squares regression (LOWESS) within each sub-grid using the default smoothing parameter of 0.33. Within-slide replicate spots analysis was used to convert the signal intensities of one replicate spot in a pair of replicate spots to the geometric mean of it and its replicate and to set the signal intensities for the other spot to zero, thus compressing the data. The within-slide replicate spots analysis also had the effect of setting the signal intensities to zero for both spots in a replicate pair to zero if the signal intensity in either channel for either spot was zero. The normalized signal intensity files thus generated in MIDAS were paired by dye-swap and checked for dye-swap consistency, again using the default parameters. Spots with greater than 2 standard deviations difference in log2 ratios between dye-swap replicates were assigned zero intensities. Spots that were within threshold consistency were averaged (geometric mean). Thus, each set of two input dye-swap files resulted in one output file. The above described operations, including setting minimum intensity cut-offs, setting signal intensities to zero for spots with signal intensities not significantly above background, averaging of within-slide replicate spots, and setting signal intensities to zero for spots with significant dye bias were standard data manipulations for array data QC and the utilization of technical replication (replicate spots and dye-swap hybridizations) to eliminate the influence of low-quality spots and/or dye bias from the downstream data analyses [57], thus leaving only biological variation to be evaluated, which was the intent of this study. Example data analysis reports for the MIDAS analyses are in Additional file 6. These analyses generated 12 result files, one from each biological replicate for each of the four life-cycle stages. Each row in the final result files corresponded to eight independent measurements (2 channels × 2 replicate spots × 2 dye-swap hybridizations). Using the TIGR MultiExperiment Viewer (MeV) software the result files were filtered to remove genes with zero intensity in more than 3 result files (percentage cut-off of 75%), leaving 10,256 spots. The genes and experiments were median centered and normalized using the default method for this software. To find spots with significant, repeatable, between-stage variation we analyzed the result files by multi-class SAM for 4 groups (amastigotes, trypomastigotes, epimastigotes, metacyclic trypomastigotes – 3 files each). The settings for the SAM analysis were: number of permutations = 1,000 (10 times the default value), select S0 using Tusher et al. method [29] (default), calculate q values? = yes (not default – slow), imputation engine = K nearest neighbors (default), number of neighbors = 10 (default), and construct hierarchical trees? = yes (not default). Significant spots were selected with a median false discovery rate (FDR) of 0.06071% and a 90th percentile FDR of 0.40582%. The microarray data were deposited in the Gene Expression Omnibus http://www.ncbi.nlm.nih.gov/geo/ under the accession GSE14641.

### qRT-PCR

Genes from functional groups of interest were selected for qRT-PCR analysis in a given life-cycle stage based on the gene being significantly regulated in the microarray analysis, as determined by SAM.

Total RNA was prepared from 3 independent samples from each life-cycle stage as described above. RNA samples were treated with DNase I (Promega) at a final concentration of 1 U/μg RNA at 37°C for 45 m. RNA was purified from the DNase digestions using RNeasy RNA purification columns (Qiagen). First strand cDNA reactions from 5 μg total RNA were primed with a mixture of oligo d(T) and a cDNA primer (Tc-18S cDNA: AAGAAATATCGGTGAACTTTCG) specific to the 3' end of *T. cruzi *18S rRNA. Briefly, 0.5 μg oligo d(T)V and 5 pmoles *T. cruzi *18S rRNA primer were added to the RNA samples and the primer/template mixtures were denatured at 65°C for 5 m. The samples were cooled to 42°C and final 20 μl reactions were assembled containing 1× first strand buffer, 10 mM DTT, 0.5 mM dNTP, 40 U RNasin, and 200 U SuperScript II reverse transcriptase. The cDNA reactions were incubated at 42°C for 1.5 h. For each RNA sample, duplicate control cDNA reactions were prepared in which the reverse transcriptase was omitted. Following first strand synthesis, the reactions were heat inactivated at 70°C for 10 m, then treated with 2 U RNase H (Invitrogen) at 37°C for 45 m.

Quantifications of selected *T. cruzi *genes were performed for stage and reference cDNA samples in duplicate on an iCyler (Bio-Rad Laboratories, Hercules, CA) with an iQ5 Multicolor Real-Time PCR Detection System (Bio-Rad). The primer sequences used in the qRT-PCR analyses are available in Additional file 7. Reactions were prepared containing 5 pmoles forward and reverse primers, 1× iQ SYBR^® ^Green Supermix (Bio-Rad), and 2 μl template DNA. Standard curves were prepared for each run using known quantities of *T. cruzi *genomic DNA (ten-fold dilutions beginning at 15 ng/μl) and primers for the gene being quantified. The raw quantifications were calculated using the iQ5 Optical Detection System software and normalized to the 18S rRNA values for each sample. The final stage/reference ratios were the averages, for each gene, of the 9 possible normalized stage/reference comparisons (3 stage samples × 3 reference samples).

## Authors' contributions

TM designed and performed the microarray and qRT-PCR experiments, analyzed and interpreted the data, and wrote the manuscript. DBW assisted in bioinformatic analyses and database creation. JA and RO helped with the microarray and proteomic data comparisons and assisted in drafting the manuscript. RT initiated and guided the project, analyzed and interpreted data, and wrote the manuscript.

## Supplementary Material

Additional file 1 **Microarray data for protein phosphatase genes significantly regulated during the life-cycle of *Trypanosoma cruzi***. The file consists of one table. Percentiles are the percentile ranks for the signal intensities within each stage. For example, a spot with a percentile of 50% in amastigotes was brighter than 50% of all of the spots in the amastigote channel. Ratios are the mean log2(stage/reference) ratios for the three biological replicates in amastigotes (AMA), trypomastigotes (TRYP), epimastigotes (EPI), and metacyclic trypomastigotes (META). Standard deviations are for the log2 ratios in the three biological replicates. Click here for file

Additional file 2 **Microarray data for protein kinase genes significantly regulated during the life-cycle of *Trypanosoma cruzi***. The file consists of one table. Percentiles are the percentile ranks for the signal intensities within each stage. For example, a spot with a percentile of 50% in amastigotes was brighter than 50% of all of the spots in the amastigote channel. Ratios are the mean log2(stage/reference) ratios for the three biological replicates in amastigotes (AMA), trypomastigotes (TRYP), epimastigotes (EPI), and metacyclic trypomastigotes (META). Standard deviations are for the log2 ratios in the three biological replicates. Click here for file

Additional file 3 **Microarray data for *Trypanosoma cruzi *genes annotated as 'integral-to-membrane' by the Gene Ontology Consortium http://www.geneontology.org significantly regulated during the life-cycle of *Trypanosoma cruzi***. The file consists of one table. Percentiles are the percentile ranks for the signal intensities within each stage. For example, a spot with a percentile of 50% in amastigotes was brighter than 50% of all of the spots in the amastigote channel. Ratios are the mean log2(stage/reference) ratios for the three biological replicates in amastigotes (AMA), trypomastigotes (TRYP), epimastigotes (EPI), and metacyclic trypomastigotes (META). Standard deviations are for the log2 ratios in the three biological replicates. Click here for file

Additional file 4 **Remapping of the array oligonucleotides to the *T. cruzi *CL Brener annotated reference genome sequence**. The file consists of one table. Shown are the results of our remapping of the Pathogen Functional Genomics Resource Center *Trypanosoma cruzi *microarray oligonucleotide sequences to the *T. cruzi *annotated genome, the original TIGR mapping, and the results of the cross-hybridization prediction. Click here for file

Additional file 5 **Gene family representation**. The file consists of one table. Shown is the representation of large gene families (cluster designations per [54]) on the Pathogen Functional Genomics Resource Center *Trypanosoma cruzi *oligonucleotide microarrays. Click here for file

Additional file 6 **Representative data analysis report**. The file consists of one figure. Sample results from filtering, normalization and dye-swap checking in the analysis of *Trypanosoma cruzi *microarray data with the Microarray Data Analysis System (MIDAS) from The Institute for Genomic Research (TIGR). Click here for file

Additional file 7 **Oligonucleotide sequences for primers used in qRT-PCR validation of *Trypanosoma cruzi *microarray data**. The file consists of one table. Click here for file

Additional file 8 **Microarray data for Figure **3, **trans-sialidases (TS) significantly regulated during the life-cycle of *Trypanosoma cruzi***. The file consists of one table. Percentiles are the percentile ranks for the signal intensities within each stage. For example, a spot with a percentile of 50% in amastigotes was brighter than 50% of all of the spots in the amastigote channel. Ratios are the mean log2(stage/reference) ratios for the three biological replicates. Standard deviations are for the log2 ratios in the three biological replicates. Click here for file

Additional file 9 **Microarray data for Figure **5A, **ribosomal protein genes significantly regulated during the life-cycle of *Trypanosoma cruzi***. The file consists of one table. Percentiles are the percentile ranks for the signal intensities within each stage. For example, a spot with a percentile of 50% in amastigotes was brighter than 50% of all of the spots in the amastigote channel. Ratios are the mean log2(stage/reference) ratios for the three biological replicates. Standard deviations are for the log2 ratios in the three biological replicates. Click here for file

Additional file 10 **Microarray data for Figure **5C, **mucin genes significantly regulated during the life-cycle of *Trypanosoma cruzi***. The file consists of one table. Percentiles are the percentile ranks for the signal intensities within each stage. For example, a spot with a percentile of 50% in amastigotes was brighter than 50% of all of the spots in the amastigote channel. Ratios are the mean log2(stage/reference) ratios for the three biological replicates. Standard deviations are for the log2 ratios in the three biological replicates. Click here for file

## References

[B1] Krieger M Freund A Avila A Muniz B Probst C Pavoni D Sunaga D Marchini F Picchi G Lenzi K Analysis of the gene expression program during the cellular differentiation of Trypanosoma cruzi (metacyclogenesis) through microarray hybridization MPM XIII: 2002 2002 Woods Hole, MA: Marine Biological Laboratory 232B

[B2] Avila AR Dallagiovanna B Yamada-Ogatta SF Monteiro-Goes V Fragoso SP Krieger MA Goldenberg S Stage-specific gene expression during Trypanosoma cruzi metacyclogenesis Genet Mol Res 2003 2 1 159 168 12917812

[B3] Minning TA Bua J Garcia GA McGraw RA Tarleton RL Microarray profiling of gene expression during trypomastigote to amastigote transition in Trypanosoma cruzi Mol Biochem Parasitol 2003 131 1 55 64 10.1016/S0166-6851(03)00189-0 12967712

[B4] Baptista CS Vencio RZ Abdala S Valadares MP Martins C de Braganca Pereira CA Zingales B DNA microarrays for comparative genomics and analysis of gene expression in Trypanosoma cruzi Mol Biochem Parasitol 2004 138 2 183 194 10.1016/j.molbiopara.2004.06.017 15555730

[B5] Paba J Ricart CA Fontes W Santana JM Teixeira AR Marchese J Williamson B Hunt T Karger BL Sousa MV Proteomic analysis of Trypanosoma cruzi developmental stages using isotope-coded affinity tag reagents Journal of proteome research 2004 3 3 517 524 10.1021/pr034075o 15253433

[B6] Paba J Santana JM Teixeira AR Fontes W Sousa MV Ricart CA Proteomic analysis of the human pathogen Trypanosoma cruzi Proteomics 2004 4 4 1052 1059 10.1002/pmic.200300637 15048986

[B7] Parodi-Talice A Duran R Arrambide N Prieto V Pineyro MD Pritsch O Cayota A Cervenansky C Robello C Proteome analysis of the causative agent of Chagas disease: Trypanosoma cruzi Int J Parasitol 2004 34 8 881 886 10.1016/j.ijpara.2004.05.002 15217726

[B8] Atwood JA 3rd Weatherly DB Minning TA Bundy B Cavola C Opperdoes FR Orlando R Tarleton RL The Trypanosoma cruzi proteome Science 2005 309 5733 473 476 10.1126/science.1110289 16020736

[B9] Atwood JA 3rd Minning T Ludolf F Nuccio A Weatherly DB Alvarez-Manilla G Tarleton R Orlando R Glycoproteomics of Trypanosoma cruzi trypomastigotes using subcellular fractionation, lectin affinity, and stable isotope labeling Journal of proteome research 2006 5 12 3376 3384 10.1021/pr060364b 17137339

[B10] Parodi-Talice A Monteiro-Goes V Arrambide N Avila AR Duran R Correa A Dallagiovanna B Cayota A Krieger M Goldenberg S Proteomic analysis of metacyclic trypomastigotes undergoing Trypanosoma cruzi metacyclogenesis J Mass Spectrom 2007 42 11 1422 1432 10.1002/jms.1267 17960573

[B11] Koumandou VL Natesan SK Sergeenko T Field MC The trypanosome transcriptome is remodelled during differentiation but displays limited responsiveness within life stages BMC genomics 2008 9 298 18573209 10.1186/1471-2164-9-298 PMC2443814

[B12] Clayton CE Life without transcriptional control? From fly to man and back again Embo J 2002 21 8 1881 1888 11953307 10.1093/emboj/21.8.1881 PMC125970

[B13] Haile S Papadopoulou B Developmental regulation of gene expression in trypanosomatid parasitic protozoa Curr Opin Microbiol 2007 10 6 569 577 10.1016/j.mib.2007.10.001 18177626

[B14] Campbell DA Thomas S Sturm NR Transcription in kinetoplastid protozoa: why be normal? Microbes Infect 2003 5 13 1231 1240 10.1016/j.micinf.2003.09.005 14623019

[B15] Jager AV De Gaudenzi JG Cassola A D'Orso I Frasch AC mRNA maturation by two-step trans-splicing/polyadenylation processing in trypanosomes Proc Natl Acad Sci USA 2007 104 7 2035 2042 17267594 10.1073/pnas.0611125104 PMC1892994

[B16] Nardelli SC Avila AR Freund A Motta MC Manhaes L de Jesus TC Schenkman S Fragoso SP Krieger MA Goldenberg S Small-subunit rRNA processome proteins are translationally regulated during differentiation of Trypanosoma cruzi Eukaryot Cell 2007 6 2 337 345 17158738 10.1128/EC.00279-06 PMC1797946

[B17] Holetz FB Correa A Avila AR Nakamura CV Krieger MA Goldenberg S Evidence of P-body-like structures in Trypanosoma cruzi Biochem Biophys Res Commun 2007 356 4 1062 1067 10.1016/j.bbrc.2007.03.104 17399688

[B18] Cassola A De Gaudenzi JG Frasch AC Recruitment of mRNAs to cytoplasmic ribonucleoprotein granules in trypanosomes Mol Microbiol 2007 65 3 655 670 10.1111/j.1365-2958.2007.05833.x 17635187

[B19] McNicoll F Muller M Cloutier S Boilard N Rochette A Dube M Papadopoulou B Distinct 3'-untranslated region elements regulate stage-specific mRNA accumulation and translation in Leishmania J Biol Chem 2005 280 42 35238 35246 10.1074/jbc.M507511200 16115874

[B20] Horn D Codon usage suggests that translational selection has a major impact on protein expression in trypanosomatids BMC genomics 2008 9 1 2 18173843 10.1186/1471-2164-9-2 PMC2217535

[B21] Duncan R DNA microarray analysis of protozoan parasite gene expression: outcomes correlate with mechanisms of regulation Trends Parasitol 2004 20 5 211 215 10.1016/j.pt.2004.02.008 15105020

[B22] Bringaud F Muller M Cerqueira GC Smith M Rochette A El-Sayed NM Papadopoulou B Ghedin E Members of a large retroposon family are determinants of post-transcriptional gene expression in Leishmania PLoS pathogens 2007 3 9 1291 1307 17907803 10.1371/journal.ppat.0030136 PMC2323293

[B23] Dallagiovanna B Correa A Probst CM Holetz F Smircich P de Aguiar AM Mansur F da Silva CV Mortara RA Garat B Functional genomic characterization of mRNAs associated with TcPUF6, a pumilio-like protein from Trypanosoma cruzi J Biol Chem 2008 283 13 8266 8273 18056709 10.1074/jbc.M703097200 PMC2276385

[B24] D'Orso I Frasch AC Functionally different AU- and G-rich cis-elements confer developmentally regulated mRNA stability in Trypanosoma cruzi by interaction with specific RNA-binding proteins J Biol Chem 2001 276 19 15783 15793 10.1074/jbc.M010959200 11278796

[B25] D'Orso I Frasch AC TcUBP-1, a developmentally regulated U-rich RNA-binding protein involved in selective mRNA destabilization in trypanosomes J Biol Chem 2001 276 37 34801 34809 10.1074/jbc.M102120200 11435421

[B26] Di Noia JM D'Orso I Sanchez DO Frasch AC AU-rich elements in the 3'-untranslated region of a new mucin-type gene family of Trypanosoma cruzi confers mRNA instability and modulates translation efficiency J Biol Chem 2000 275 14 10218 10227 10.1074/jbc.275.14.10218 10744707

[B27] Robles A Clayton C Regulation of an amino acid transporter mRNA in Trypanosoma brucei Mol Biochem Parasitol 2008 157 1 102 106 10.1016/j.molbiopara.2007.09.005 17996963

[B28] Noe G De Gaudenzi JG Frasch AC Functionally related transcripts have common RNA motifs for specific RNA-binding proteins in trypanosomes BMC molecular biology 2008 9 1 107 19063746 10.1186/1471-2199-9-107 PMC2637893

[B29] Tusher VG Tibshirani R Chu G Significance analysis of microarrays applied to the ionizing radiation response Proc Natl Acad Sci USA 2001 98 9 5116 5121 11309499 10.1073/pnas.091062498 PMC33173

[B30] Yoshida N Molecular basis of mammalian cell invasion by Trypanosoma cruzi An Acad Bras Cienc 2006 78 1 87 111 16532210 10.1590/s0001-37652006000100010

[B31] Huang da W Sherman BT Lempicki RA Systematic and integrative analysis of large gene lists using DAVID bioinformatics resources Nature protocols 2009 4 1 44 57 10.1038/nprot.2008.211 19131956

[B32] Dennis G Jr Sherman BT Hosack DA Yang J Gao W Lane HC Lempicki RA DAVID: Database for Annotation, Visualization, and Integrated Discovery Genome biology 2003 4 5 P3 10.1186/gb-2003-4-5-p3 12734009 PMC193660

[B33] Brandao A Urmenyi T Rondinelli E Gonzalez A de Miranda AB Degrave W Identification of transcribed sequences (ESTs) in the Trypanosoma cruzi genome project Mem Inst Oswaldo Cruz 1997 92 6 863 866 10.1590/S0074-02761997000600024 9580492

[B34] Buscaglia CA Campo VA Frasch AC Di Noia JM Trypanosoma cruzi surface mucins: host-dependent coat diversity Nature reviews 2006 4 3 229 236 10.1038/nrmicro1351 16489349

[B35] Aebersold R Mann M Mass spectrometry-based proteomics Nature 2003 422 6928 198 207 10.1038/nature01511 12634793

[B36] Josic D Clifton JG Mammalian plasma membrane proteomics Proteomics 2007 7 16 3010 3029 10.1002/pmic.200700139 17654460

[B37] Teixeira SM Russell DG Kirchhoff LV Donelson JE A differentially expressed gene family encoding "amastin," a surface protein of Trypanosoma cruzi amastigotes J Biol Chem 1994 269 32 20509 20516 8051148

[B38] Ohno S Evolution by gene duplication 1970 New York,: Springer

[B39] Diehl S Diehl F El-Sayed NM Clayton C Hoheisel JD Analysis of stage-specific gene expression in the bloodstream and the procyclic form of Trypanosoma brucei using a genomic DNA-microarray Mol Biochem Parasitol 2002 123 2 115 123 10.1016/S0166-6851(02)00138-X 12270627

[B40] Luu VD Brems S Hoheisel JD Burchmore R Guilbride DL Clayton C Functional analysis of Trypanosoma brucei PUF1 Mol Biochem Parasitol 2006 150 2 340 349 10.1016/j.molbiopara.2006.09.007 17052765

[B41] Cohen-Freue G Holzer TR Forney JD McMaster WR Global gene expression in Leishmania Int J Parasitol 2007 37 10 1077 1086 10.1016/j.ijpara.2007.04.011 17574557

[B42] Saxena A Worthey EA Yan S Leland A Stuart KD Myler PJ Evaluation of differential gene expression in Leishmania major Friedlin procyclics and metacyclics using DNA microarray analysis Mol Biochem Parasitol 2003 129 1 103 114 10.1016/S0166-6851(03)00100-2 12798511

[B43] Rochette A Raymond F Ubeda JM Smith M Messier N Boisvert S Rigault P Corbeil J Ouellette M Papadopoulou B Genome-wide gene expression profiling analysis of Leishmania major and Leishmania infantum developmental stages reveals substantial differences between the two species BMC genomics 2008 9 255 18510761 10.1186/1471-2164-9-255 PMC2453527

[B44] De Gaudenzi J Frasch AC Clayton C RNA-binding domain proteins in Kinetoplastids: a comparative analysis Eukaryot Cell 2005 4 12 2106 2114 16339728 10.1128/EC.4.12.2106-2114.2005 PMC1317496

[B45] Tarleton RL Koller BH Latour A Postan M Susceptibility of beta 2-microglobulin-deficient mice to Trypanosoma cruzi infection Nature 1992 356 6367 338 340 10.1038/356338a0 1549177

[B46] Abrahamsohn IA Cytokines in innate and acquired immunity to Trypanosoma cruzi infection Braz J Med Biol Res 1998 31 1 117 121 10.1590/S0100-879X1998000100015 9686187

[B47] Fichera LE Albareda MC Laucella SA Postan M Intracellular growth of Trypanosoma cruzi in cardiac myocytes is inhibited by cytokine-induced nitric oxide release Infect Immun 2004 72 1 359 363 14688116 10.1128/IAI.72.1.359-363.2004 PMC343980

[B48] Li WH Yang J Gu X Expression divergence between duplicate genes Trends Genet 2005 21 11 602 607 10.1016/j.tig.2005.08.006 16140417

[B49] Piras R Piras MM Henriquez D The effect of inhibitors of macromolecular biosynthesis on the in vitro infectivity and morphology of Trypanosoma cruzi trypomastigotes Mol Biochem Parasitol 1982 6 2 83 92 10.1016/0166-6851(82)90067-6 6752706

[B50] Tomlinson S Vandekerckhove F Frevert U Nussenzweig V The induction of Trypanosoma cruzi trypomastigote to amastigote transformation by low pH Parasitology 1995 110 547 554 10.1017/S0031182000065264 7541124

[B51] Rondinelli E Silva R Carvalho JF de Almeida Soares CM de Carvalho EF de Castro FT Trypanosoma cruzi: an in vitro cycle of cell differentiation in axenic culture Exp Parasitol 1988 66 2 197 204 10.1016/0014-4894(88)90091-4 3294026

[B52] Isola EL Lammel EM Gonzalez Cappa SM Trypanosoma cruzi: differentiation after interaction of epimastigotes and Triatoma infestans intestinal homogenate Exp Parasitol 1986 62 3 329 335 10.1016/0014-4894(86)90039-1 3023131

[B53] Sturm NR Vargas NS Westenberger SJ Zingales B Campbell DA Evidence for multiple hybrid groups in Trypanosoma cruzi Int J Parasitol 2003 33 3 269 279 10.1016/S0020-7519(02)00264-3 12670512

[B54] El-Sayed NM Myler PJ Bartholomeu DC Nilsson D Aggarwal G Tran AN Ghedin E Worthey EA Delcher AL Blandin G The genome sequence of Trypanosoma cruzi, etiologic agent of Chagas disease Science 2005 309 5733 409 415 10.1126/science.1112631 16020725

[B55] Obado SO Taylor MC Wilkinson SR Bromley EV Kelly JM Functional mapping of a trypanosome centromere by chromosome fragmentation identifies a 16-kb GC-rich transcriptional "strand-switch" domain as a major feature Genome research 2005 15 1 36 43 15632088 10.1101/gr.2895105 PMC540271

[B56] Liao P Chen T Chung P A fast algorithm for multilevel thresholding Journal of Information Science and Engineering 2001 17 713 727

[B57] Quackenbush J Microarray data normalization and transformation Nature genetics 2002 32 Suppl 496 501 10.1038/ng1032 12454644

[B58] Altschul SF Gish W Miller W Myers EW Lipman DJ Basic local alignment search tool J Mol Biol 1990 215 3 403 410 2231712 10.1016/S0022-2836(05)80360-2

