# Supplementary figures and images for: The steady-state transcriptome of the four major life-cycle stages of Trypanosoma cruzi
Source: BMC Genomics. 2009 Aug 7;10:370. doi: 10.1186/1471-2164-10-370 (PMC2907688; doi:10.1186/1471-2164-10-370)

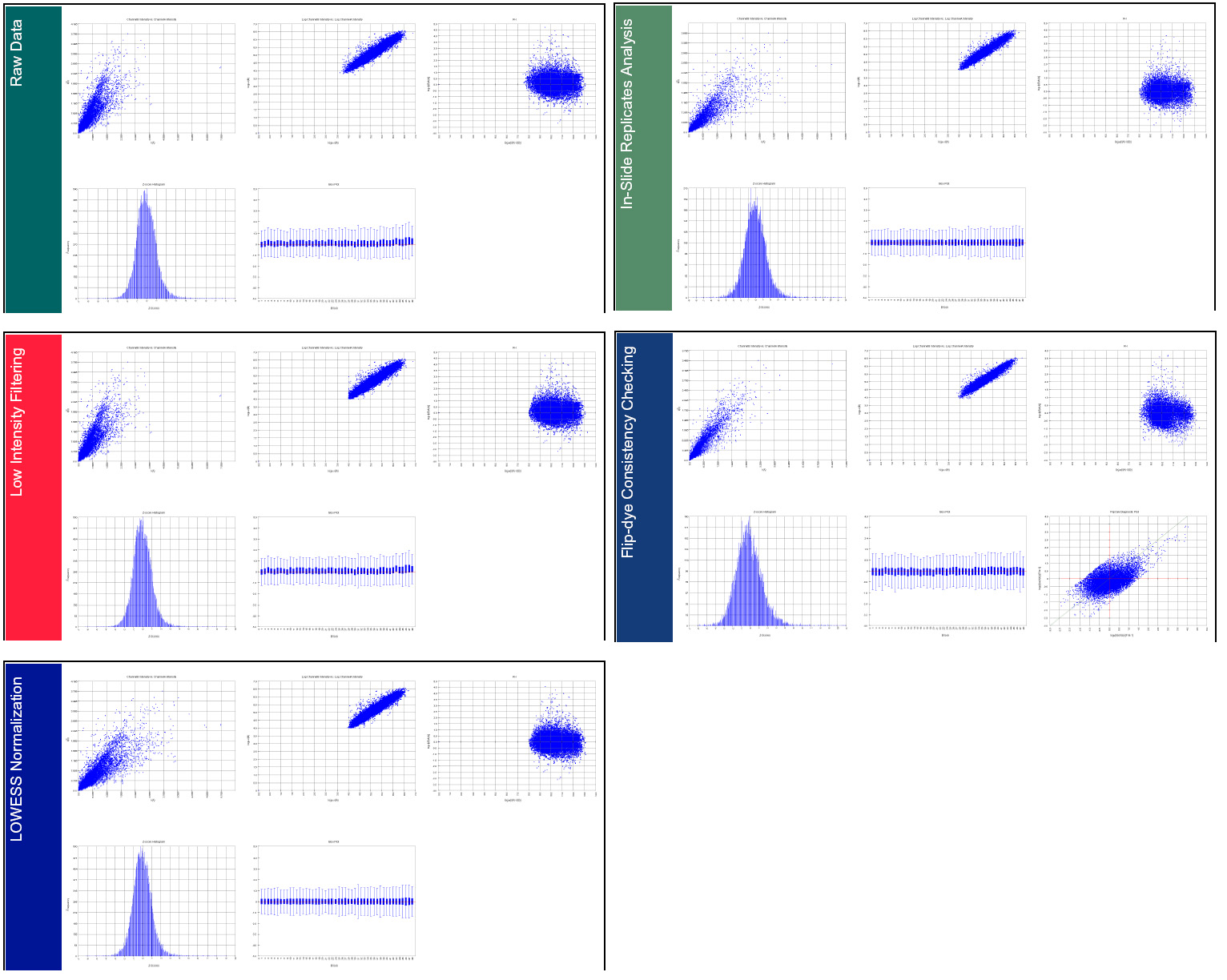

Supplement: Additional file 6 — Representative data analysis report. The file consists of one figure. Sample results from filtering, normalization and dye-swap checking in the analysis of Trypanosoma cruzi microarray data with the Microarray Data Analysis System (MIDAS) from The Institute for Genomic Research (TIGR). [file 1471-2164-10-370-S6.jpeg]
